# Moving Towards Intelligent Transportation via Artificial Intelligence and Internet-of-Things

**DOI:** 10.3390/s20236945

**Published:** 2020-12-04

**Authors:** Miltiadis D. Lytras, Kwok Tai Chui, Ryan Wen Liu

**Affiliations:** 1 King Abdulaziz University, Jeddah P.O. Box 34689, Saudi Arabia; mlytras@acg.edu; 2 Effat College of Engineering, Effat University, Jeddah P.O. Box 34689, Saudi Arabia; 3 Department of Technology, School of Science and Technology, The Open University of Hong Kong, Hong Kong, China; 4 Hubei Key Laboratory of Inland Shipping Technology, School of Navigation, Wuhan University of Technology, Wuhan 430063, China; wenliu@whut.edu.cn

One of the key smart city visions is to bring smarter transport networks, specifically intelligent/smart transportation. It facilitates safe and effective physical movement and interaction of humans, animals, and goods. Typical global issues include sustainable energy, traffic accidents, traffic congestion, logistic management, data analysis, security, and privacy. In recent years, the internet-of-things (IoT) and artificial intelligence (AI) have taken a leading role in achieving this smart city vision. The former provides a solid infrastructure for scalable and robust data collection and transmission. The latter brings creative and innovative elements to machines for intelligent transportation applications. In this special issue, “Internet of Things and Artificial Intelligence in Transportation Revolution”, ten (10) research articles have been published. These articles generate a meaningful discussion around the impacts of AI and IoT in intelligent transportation. This editorial not only summarizes the special issue articles but also shares other hot research topics.

Transportation plays an essential role in today’s economic and social development. As daily road users, we need to ensure safe and effective travel. According to the World Health Organization (WHO), annual road traffic deaths and injuries reach 1.35 million and 50 million, respectively [1]. Based on Web of Science statistics, there has been increasing attention on intelligent/smart transportation since 2007, reflected by the rising number of research publications. The average percentage increase in the number of research publications is 51.6% from 2014 to 2019.

The first article, “Decision-making for the autonomous navigation of maritime autonomous surface ships based on scene division and deep reinforcement learning” [2] authored by X. Zhang, C. Wang, Y. Liu, and X. Chen, considered maritime autonomous surface ships (MASSs). Attention has been drawn to adaptive navigation and an uncertain environment. An artificial potential field-deep reinforcement learning approach was proposed. Their experiment revealed that the proposed method significantly reduced the collision rate from 2.24% to 1.16%, compared with the traditional deep reinforcement learning approach.

Y. Jiang, B. Liu, Z. Wang, and X. Yi presented an article “Start from scratch: A crowdsourcing-based data fusion approach to support location-aware applications” [3]. Multi-dimensional crowdsourcing with multi-resolution ambient map and trade coding has been applied to the indoor localization problem. Twenty-six volunteers participated in the data collection process. In total, 931 crowdsourcing data traces were collected from five types of smartphones on two floors with a total floor space of 4000 m^2^. Results showed that half of the data points were perfect, whereas 90% of the data points were deviations by two cells.

In [4], G. Baldini, F. Geib, and R. Giuliani published an article “Continuous authentication of automotive vehicles using inertial measurement units.” It was about the continuous authentication of automotive vehicles using inertial measurement units. The workflows can be summarized as (i) data synchronization; (ii) laps extraction; (iii) segmentation; (iv) normalization; (v) feature extraction; (vi) construction of machine learning models including K-nearest neighbors, decision tree, random forest, AdaBoost, and support vector machine. The accuracy ranged from 85% (decision tree) to 90% (support vector machine).

Optimization of vehicle arrival time and signal timings is vital for traffic signal control. W. Wu, L. Huang, R. Du, presented an article “Simultaneous optimization of vehicle arrival time and signal timings within a connected vehicle environment” [5]. A time-based sliding window approach was applied to solve the optimization problem. An experiment was designed with two cases, whereby the proposed algorithm significantly reduced the number of stops by 29–77.5% and the average vehicle delay by 37.8–54%. Analysis has revealed the feasibility of the proposed model in varying communication distance, the market penetration of connected vehicles, and speed guidance’s compliance rate.

In [6], S. Guo, X. Zhang, Y. Zheng, and Y. Du focused on path planning of unmanned ships in their article “An Autonomous Path Planning Model for Unmanned Ships Based on Deep Reinforcement Learning.” The researchers proposed an artificial potential field based deep deterministic policy gradient approach for path planning of unmanned ships in the unknown environment. It reduced the total iteration time from 339 s to 395 s, the optimal decision time from 282 s to 236 s, convergence steps from 133 to 68, and the number of collisions from 72 to 63, compared with a traditional deep deterministic policy gradient.

Another work, “A generic design of driver drowsiness and stress recognition using MOGA optimized deep MKL-SVM” authored by K. T. Chui, M. D. Lytras, and R. W. Liu [7], presented an approach based on a multiobjective genetic algorithm and multiple kernel learning based support vector machine. It could be applied to both driver drowsiness and stress recognition. The sensitivity, specificity, and area under the receiver operating characteristic curve were 99%, 98.3%, and 97.1%, respectively, for driver drowsiness recognition. On the other hand, they were 98.7%, 98.4%, and 96.9%, respectively, for driver stress recognition.

Y. Guo, B. Li, M. D. Christie, Z. Li, M. A. Sotelo, Y. Ma, and Z. Li published an article “Hybrid dynamic traffic model for freeway flow analysis using a switched reduced-order unknown-input state observer” [8]. Dynamic graph hybrid automata with a cell transmission model was proposed for vehicle density estimation. The experimental environment has been divided into 10 cells. Traffic counting sensors were installed in 7 cells. The estimated density was close to the real density in most of the cells. The performance would be degraded when traffic flow on the main road was being affected by on-ramp vehicles.

In [9], an article “Using Deep Learning to Forecast Maritime Vessel Flows” was shared by X. Zhou, Z. Liu, F. Wang, Y. Xie, X. Zhang. A bidirectional long short-term memory network with a convolutional neural network was proposed for maritime vessel flows forecast. Four cases have been studied. Results indicated the proposed algorithm achieved the lowest error rate (20–22.5%), compared with a support vector regression (50.1–51.4%), standalone convolution neural network (24–26%), and long short-term memory (21–24%).

The article “Vision-Based Machine Learning Method for Barrier Access Control Using Vehicle License Plate Authentication” published in this special issue is authored by K. T. Islam, R. G. Raj, S. M. Shamsul Islam, S. Wijewickrema, M. S. Hossain, T. Razmovski, S. A. O’Leary [10]. An artificial neural network achieved vehicle license plate recognition. Both synthetic and real data are applied for the performance evaluation of the proposed method. The proposed algorithm statistically outperformed existing works. The reported accuracy was excellent (99.7%).

Finally, G. Baldini, R. Giuliani, and F. Geib presented an article “On the application of time frequency convolutional neural networks to road anomalies identification with accelerometers and gyroscopes” [11]. It proposed a convolutional neural network approach to detect road anomalies from data collected by an inertial measurement unit. The achieved accuracy was 97.2%. The research implication relates to the benefits of management by the road infrastructure team concerning the monitoring of road surface quality. Moreover, it helps in improving the accuracy of autonomous vehicle positioning.

In 2015, all United Nations member states agreed to build strong partnerships to meet 17 sustainable development goals (SDGs) and 169 targets [12]. Among all targets, some of them are related to intelligent transportation, including SDG Goal 3 Target 3.6, SDG Goal 7, SDG Goal 8, SDG Goal 11 Target 11.2, SDG Goal 12 Target 12.C, and SDG Goal 14.

Various emerging key applications are suggested for exploration. A study [13] has applied artificial intelligence and the internet-of-things to enhance energy’s cleanliness and affordability. The work [14] has shared the use case of a 6G-enabled maritime IoT system. Parallel computing and cloud computing techniques have become proper options for high-performance computing services [15,16]. The computing services can be moved locally via edge computing [17] and fog computing [18]. The ultimate vision is a new integrated eco-system of value-adding services capable of merging the human semantic [19] and social web [20]. It will have enormous capabilities for sophisticated knowledge and data creation [21] for the dynamic composition of value-adding services in various domains, e.g., transportation [2,3,4,5,6,7,8,9,10,11,12,13,14,15,16,17,18] and healthcare [22]. A new revolution in computing is here to stay with a focus on cyber-physical systems. 

The guest editors would like to thank the contributions of all colleagues and reviewers. We are grateful for your support. 
